# Serotonin transporter deficiency drives estrogen-dependent obesity and glucose intolerance

**DOI:** 10.1038/s41598-017-01291-5

**Published:** 2017-04-25

**Authors:** Weibin Zha, Horace T. B. Ho, Tao Hu, Mary F. Hebert, Joanne Wang

**Affiliations:** 1 Department of Pharmaceutics, University of Washington, Seattle, Washington USA; 2 Department of Pharmacy, University of Washington, Seattle, WA USA; 3 Department of Obstetrics & Gynecology, University of Washington, Seattle, WA USA; 4 Nutrition Obesity Research Center, University of Washington, Seattle, WA USA

## Abstract

Depression and use of antidepressant medications are both associated with increased risk of obesity, potentially attributed to a reduced serotonin transporter (SERT) function. However, how SERT deficiency promotes obesity is unknown. Here, we demonstrated that *SERT* ^−/−^ mice display abnormal fat accumulation in both white and brown adipose tissues, glucose intolerance and insulin resistance while exhibiting suppressed aromatase (Cyp19a1) expression and reduced circulating 17β-estradiol levels. 17β-estradiol replacement in *SERT* ^−/−^ mice reversed the obesity and glucose intolerance, supporting a role for estrogen in SERT deficiency-associated obesity and glucose intolerance. Treatment of wild type mice with paroxetine, a chemical inhibitor of SERT, also resulted in Cyp19a1 suppression, decreased circulating 17β-estradiol levels, abnormal fat accumulation, and glucose intolerance. Such effects were not observed in paroxetine-treated *SERT* ^−/−^ mice. Conversely, pregnant *SERT* ^−/−^ mice displayed normalized estrogen levels, markedly reduced fat accumulation, and improved glucose tolerance, which can be eliminated by an antagonist of estrogen receptor α (ERα). Together, these findings support that estrogen suppression is involved in SERT deficiency-induced obesity and glucose intolerance, and suggest approaches to restore 17β-estradiol levels as a novel treatment option for SERT deficiency associated obesity and metabolic abnormalities.

## Introduction

Depression and obesity are highly prevalent problems in the world and associated with various health complications^1, 2^. Depression and obesity often co-occur and their association has been presumed and repeatedly reported^3, 4^. There is growing evidence that antidepressant treatment is also associated with obesity^5, 6^. Yet causes underlying these associations are largely unclear. Serotonin (5-hydroxytryptamine, 5-HT) transporter (SERT) could be a critical link between depression and obesity, as it is well known that SERT is the major therapeutic target of a large class of antidepressant drugs, and also plays a vital role in the regulation of body fat stores and glucose homeostasis^7^. Genetic deletion of SERT has been reported to lead to obesity and impaired glucose tolerance^8^, and there is also increasing clinical recognition that low SERT expression is linked with increased risk of weight gain and type 2 diabetes^9–11^. However, it is unknown how SERT deficiency promotes obesity and impaired glucose tolerance.

5-HT is a key neurotransmitter in appetite regulation in the brain, and direct depletion in mice leads to marked hyperphagia and obesity^12^. However, the central effect of 5-HT on appetite regulation likely has limited involvement in the development of obesity caused by SERT deficiency. SERT is directly involved in 5-HT signaling pathway as it re-uptakes the molecule^7^. SERT-deficient mice exhibit an increase of extracellular 5-HT concentrations in the brain^13^ and demonstrate reduced food intake^8^. However, these mice developed an obese phenotype under normal diet^7^, suggesting an involvement of propensity to obesity in the peripheral tissue rather than central 5-HT functions on appetite control. Peripheral 5-HT is produced by tryptophan hydroxylase-1 (Tph1), which has been shown to affect adipose tissue function and energy balance^14, 15^. Mice deficient in both SERT and Tph1 have markedly depleted 5-HT levels in peripheral tissues^16, 17^. However, *Tph1* ^*−/−*^ mice fed on high-fat diet are protected from obesity and insulin resistance^15^, which is opposite to the obese phenotype of SERT knockouts. These observations suggest that peripheral 5-HT functions may not be directly involved in the development of obesity and glucose intolerance in *SERT* ^*−/−*^ mice. Therefore, many questions remain regarding the mechanisms underlying SERT deficiency-associated obesity and glucose intolerance.

Estrogen is known to play an important role in regulating adipose tissue distribution, energy expenditure and glucose homeostasis^18^. In women, estrogen deficiency at menopause is associated with an increased propensity of obesity and risk for the development of type 2 diabetes^19^. In experimental animals, reduction of circulating estrogen levels by ovariectomy results in obesity, which can be reversed by estrogen replacement^20^. Genetic analyses in mice also demonstrated that lack of 17β-estradiol synthesis by targeted disruption of the aromatase gene (*Cyp19a1*) leads to a progressive increase in adiposity without hyperphagia^21^. Lastly, estrogenic mediation of serotonergic systems in depressive disorders has been documented extensively^22–24^. However, it is unknown whether estrogen and the serotonergic system interact in the regulation of obesity. We hypothesized that estrogen suppression may contribute to SERT deficiency-induced obesity and glucose intolerance. This possibility is important, because if true, approaches to restore 17β-estradiol levels may retard obesity and glucose intolerance caused by SERT deficiency.

In the current study, we utilized a SERT knockout mouse model, SSRI treatment, estrogen replacement, and pregnancy-induced hormonal changes to clarify the role of estrogen in the development of SERT-related obesity and metabolic abnormalities. We found that SERT deletion or pharmacological inhibition suppresses ovarian Cyp19a1 expression, reducing circulating 17β-estradiol levels, leading to abnormal fat accumulation, insulin resistance, and glucose intolerance. These metabolic effects can be completely alleviated with 17β-estradiol treatment or reversed by pregnancy. In addition, we provide evidence that 17β-estradiol action is dependent on the estrogen receptor α (ERα) signaling pathway. Our findings provide direct *in vivo* evidence for the involvement of estrogen suppression in SERT deficiency-induced obesity and glucose intolerance and suggest therapeutic implications for SERT deficiency-associated metabolic abnormalities.

## Results

### Female *SERT*^−/−^ mice exhibit increased visceral adiposity and brown fat lipoatrophy

**Figure 1:**
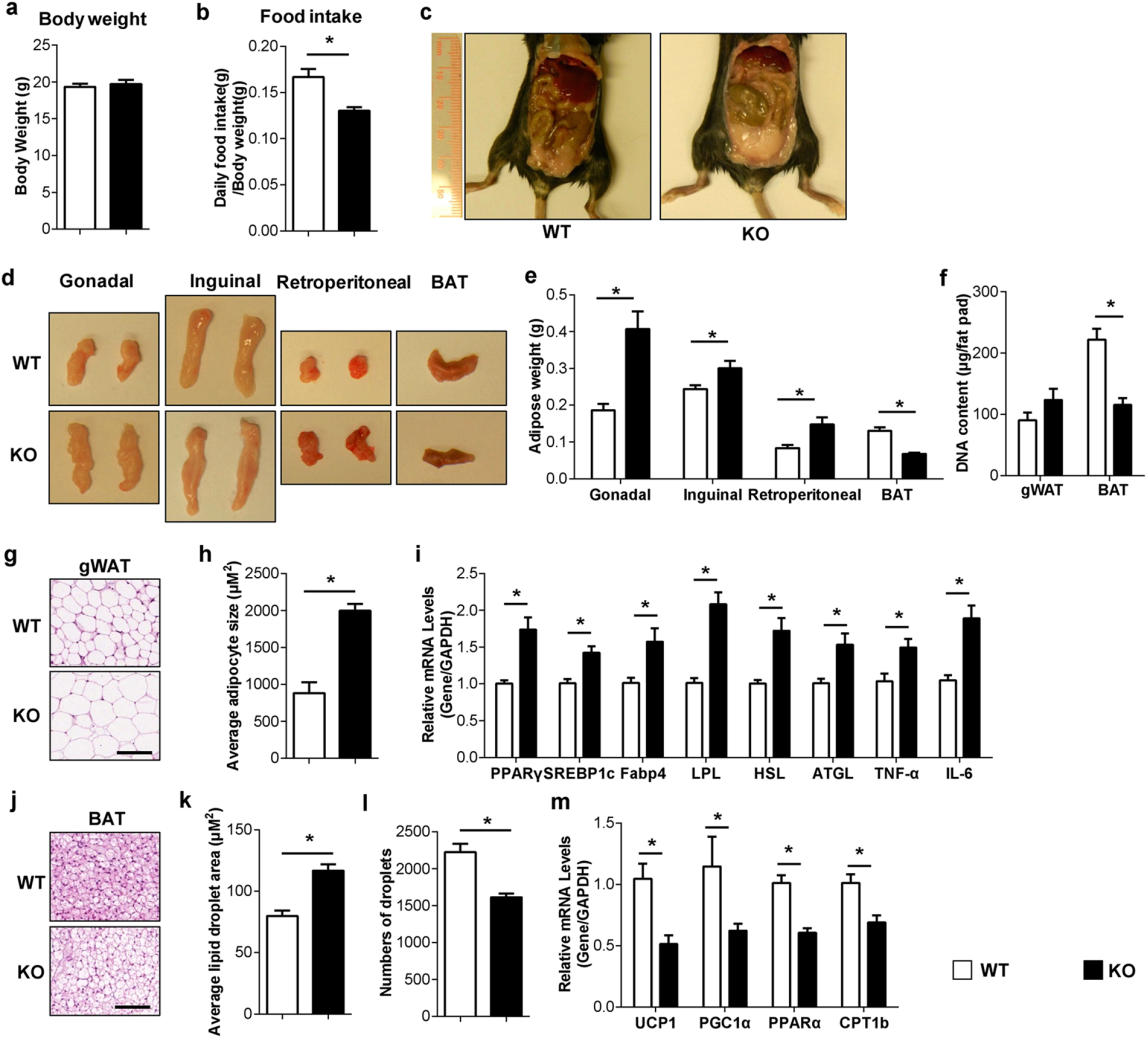
SERT deficiency leads to increased visceral adiposity and brown fat lipoatrophy in female mice. (**a–c**) Body weight, food intake and morphologic findings of WT and *SERT* ^−/−^ female mice at 3-month (n = 9–10 per group). (**d** and **e**) Representative fat tissues and weights at time of sacrifice (n = 6–12 per group). (**f**) Genomic DNA content per fat depot (n = 6–7 per group). (**g**) Representative images of H&E-stained gonadal white adipose tissue (gWAT) sections (Scale bars: 100 µm). (**h**) Average adipocyte size per 10× field quantified using ImageJ. (**i**) Gene expression analysis in gWAT by qRT-PCR (n = 6–7 per group). (**j**) Representative images of H&E-stained brown adipose tissue (BAT) sections (Scale bars: 100 µm). (**k** and **l**) Average lipid droplet area and number per 20× field in BAT was quantified using ImageJ. (**m**) Gene expression analysis in BAT by qRT-PCR (n = 6–7 per group). *P < 0.05. Values are reported as mean ± SEM.

A previous study reported reduced food intake but increased body weight and fat content in male *SERT* ^−/−^ mice under both normal and high fat diets^8^. To examine if such phenotypes exist in female mice and to further evaluate adipose tissue mass and cellularity, we analyzed and compared body weight, food intake, white and brown tissue depots in female wild type (WT) and *SERT* ^−/−^ mice. Female mice lacking SERT showed no difference in body weight and reduced food intake compared with wild-type (WT) mice at three months (Fig. 1a,b). However, upon dissection, female *SERT* ^−/−^ mice exhibited a significant increase in white adipose tissue (WAT) depots (Fig. 1c–e) and enlarged adipocytes (Fig. 1g,h). Increased WAT weights and enlarged adipocytes without body weight change were also observed in another cohort of 6-month old female WT and *SERT* ^−/−^ mice (Supplementary Fig. 1a–e). In addition, genomic DNA content per depot showed no significant difference between *SERT* ^−/−^ and WT mice (Fig. 1f), suggesting that the expansion of WAT mass is due to an increase in cell size rather than cell number. We then analyzed the expression of the genes involved in fat synthesis (PPARγ, SREBP1c, Fabp4, and LPL) and triglyceride breakdown (HSL and ATGL). Compared with WT mice, *SERT* ^−/−^ mice showed increased PPARγ, SREBP1c, Fabp4, LPL, HSL and ATGL mRNA levels (Fig. 1i). Furthermore, adipose IL-6 and TNF-α mRNA levels (Fig. 1i) were significantly higher in *SERT* ^−/−^ adipose compared with WT, indicating that lipid accumulation in *SERT* ^−/−^ mice stimulated adipose inflammation.

In contrast to WAT expansion, there was a reduction of brown adipose tissue (BAT) mass in female *SERT* ^−/−^ mice (Fig. 1d,e). BAT depots from *SERT* ^−/−^ mice had a lower degree of cellularity (Fig. 1f), and contained a higher amount of unilocular fat droplets (Fig. 1j). This was further confirmed by analysis of lipid droplet area and number (Fig. 1k,l), demonstrating that the average area of lipid droplets was greatly increased in female *SERT* ^−/−^ mice compared to WT controls. Reduction of BAT mass (Supplementary Fig. 1b,c) and higher amount of lipid content in brown fat (Supplementary Fig. 1f–h) were also observed in 6-month old female *SERT* ^−/−^ mice. The abnormal lipid accumulation in BAT is often accompanied by changes in UCP1 expression, a key regulator of thermogenesis^25^. Consistent with more lipid accumulation in BAT of *SERT* ^−/−^ mice, BAT UCP1 mRNA expression was clearly reduced (Fig. 1m). Interestingly, we also observed reduction of PGC1α, PPARα, and CPT1b gene expressions related to fatty acid oxidation in female *SERT* ^−/−^ mice (Fig. 1m), indicating compromised oxidative responses in *SERT* ^−/−^ mice. Taken together, these data demonstrate that SERT deficiency increased fat accumulation in BAT and impaired BAT function, which may also contribute to adiposity in female *SERT* ^−/−^ mice.

### Female *SERT*^−/−^ mice show glucose intolerance and insulin resistance

**Figure 2:**
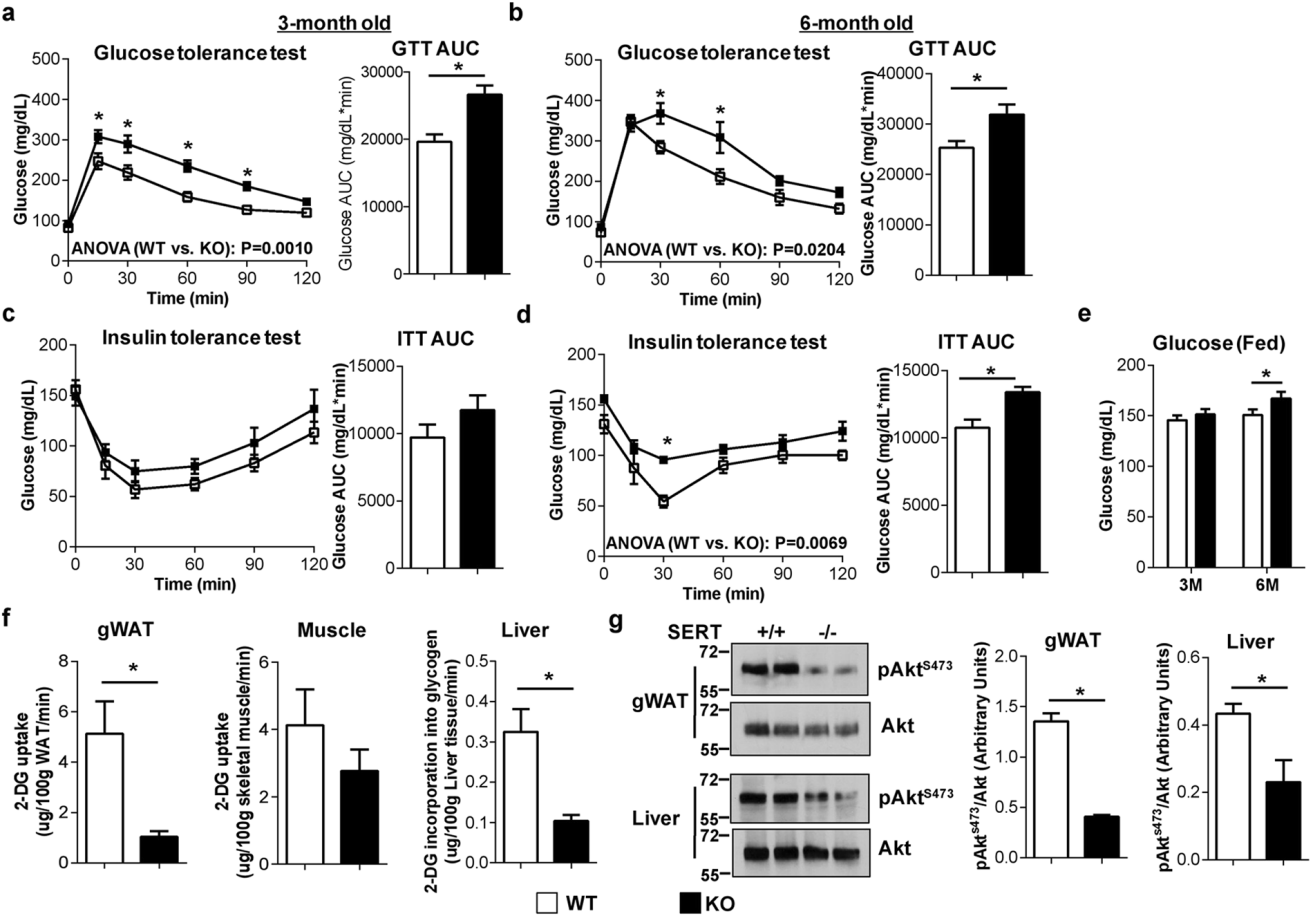
SERT deficiency impairs glucose homeostasis in female mice. (**a**–**d**) Glucose tolerance test (GTT) (16 hours of fasting) and insulin tolerance test (ITT) (6 hours of fasting) were performed in WT and *SERT* ^−/−^ female mice at 3-month (**a** and **c**) and 6-month age (**b** and **d**) (n = 6–11 per group). The repeated measures ANOVA P value is provided. The corresponding GTT AUC and ITT AUC were calculated. (**e**) Fed blood glucose levels were measured (n = 6–10 per group). (**f**) *In vivo* glucose uptake. 3-month-old WT and *SERT* ^−/−^ female mice were fasted overnight and injected i.p. with a mixture of glucose and [^3^H]2-DG, and the accumulation of the 2-DG in gWAT, skeletal muscle (gastrocnemius) and liver were determined (n = 6–10 per group). (**g**) Akt^S473^ phosphorylation relative to total Akt in gonadal WAT and liver from WT and *SERT* ^−/−^ female mice (3-month old) euthanized 10 min following an injection of 0.75 U kg^−1^ insulin (n = 3 per group). Uncut blots are included in the Supplementary information. *P < 0.05. Values are reported as mean ± SEM.

Due to the well-established role of abnormal fat deposition in the development of insulin resistance^26, 27^, we investigated glucose and insulin response in female *SERT* ^−/−^ mice. Under normal diet, female *SERT* ^−/−^ mice showed glucose intolerance at the age as early as 3 months and persisted at 6 months (Fig. 2a,b). Insulin resistance (Fig. 2d), increased fasting (Fig. 2b; time 0) and fed (Fig. 2e) blood glucose levels were observed in 6-month old female, but not 3-month old, female *SERT* ^−/−^ mice (Fig. 2c,e). To determine which insulin target tissues were responsible for the impaired glucose homeostasis, mice were injected with [^3^H]2-deoxyglucose ([^3^H]2-DG) in combination with glucose (2 g/kg body weight)^28^. In female *SERT* ^−/−^ mice, 2-DG uptake in gonadal WAT (gWAT) was significantly reduced, and uptake in skeletal muscle also showed a trend towards reduction (Fig. 2f). Incorporation into liver glycogen was also reduced (Fig. 2f). Consistent with the reduction in glucose uptake, impaired insulin/Akt signaling was observed in gWAT and liver obtained from female *SERT* ^−/−^ mice after insulin injection (Fig. 2g). The reduced glucose disposal in association with impaired Akt signaling strongly suggests that the signaling pathways involved in insulin regulation of glucose disposal in WAT and liver are desensitized in female *SERT* ^−/−^ mice.

### Estrogen suppression underlies abnormal fat accumulation and insulin resistance in *SERT*^−/−^ mice

**Figure 3:**
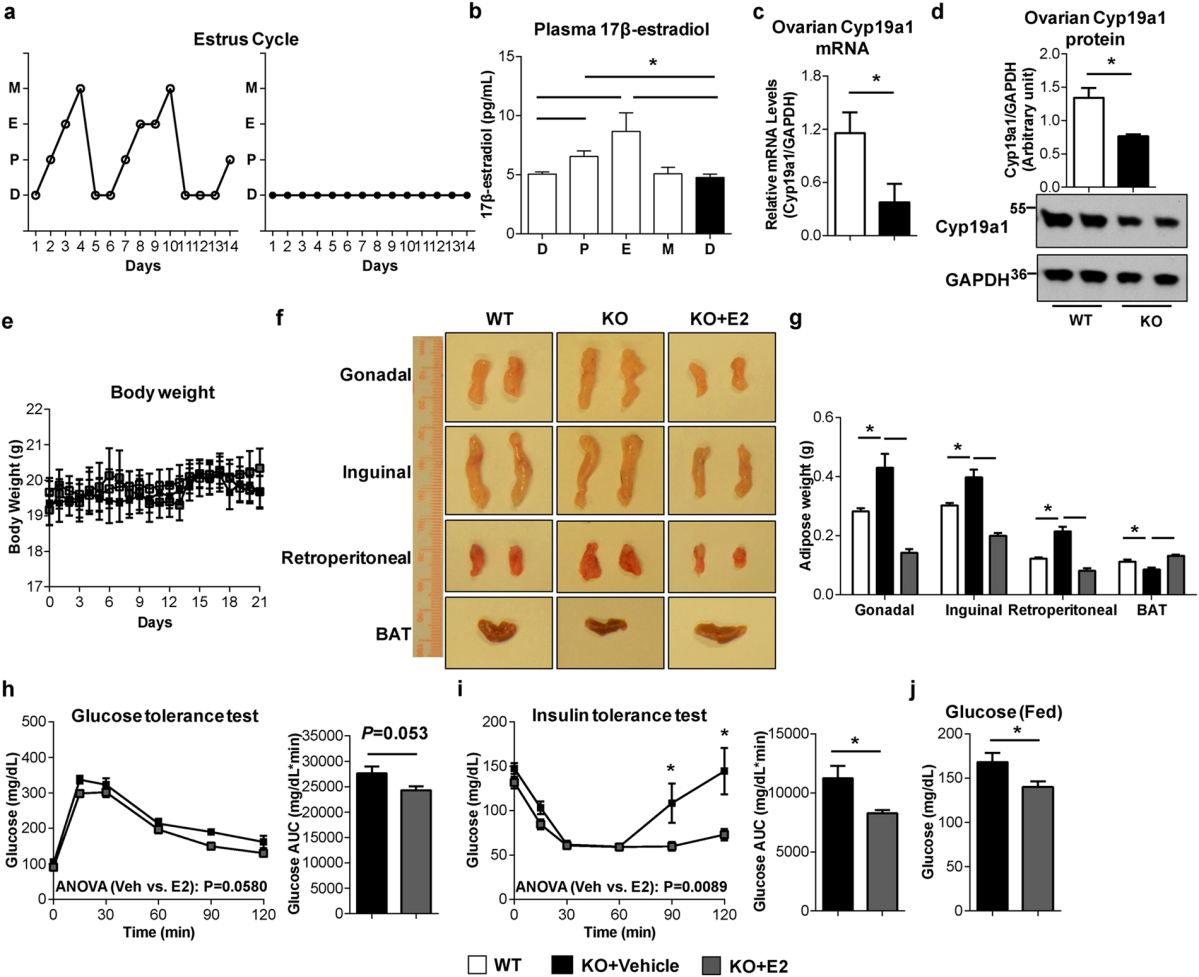
The reduction of estrogen levels contributes to abnormal fat accumulation and insulin resistance in *SERT* ^−/−^ female mice. (**a**) Representative estrous cycle stage profiles of WT and *SERT* ^−/−^ female mice. (**b**) Plasma 17β-estradiol levels of WT and *SERT* ^−/−^ female mice at 3-month age (n = 6 per group). (**c**) Cyp19a1 mRNA expression in ovary was quantified by RT-qPCR, normalized to GAPDH, and expressed relative to the control group (n = 5–6 per group). (**d**) Cyp19a1 protein levels in ovary were quantified by western blotting (n = 4 per group). Uncut blots are included in the Supplementary information. (**e**) Body weight of WT mice and *SERT* ^−/−^ female mice treated with placebo or 17β-estradiol for 3 weeks (n = 5–6 per group). (**f** and **g**) Representative fat tissues and weight at time of sacrifice (n = 5–6 per group). (**h** and **i**) GTT and ITT were performed on *SERT* ^−/−^ female mice treated with placebo or 17β-estradiol (n = 5–6 per group). The repeated measures ANOVA P value is provided. The corresponding GTT AUC and ITT AUC were calculated. (**j**) Fed blood glucose levels were measured (n = 5–6 per group). *P < 0.05. Values are reported as mean ± SEM.

Female *SERT* ^−/−^ mice showed increased fat mass, adipocyte size, insulin resistance and glucose intolerance with minimal body weight changes. These phenotypes are remarkably similar to those reported for estrogen deficiency^21^. To test if circulating 17β-estradiol is affected, we first analyzed the estrous cycles in WT and *SERT* ^−/−^ female mice as estrogen levels are known to fluctuate with the reproductive cycle. Female WT mice exhibited a normal 4- to 5-day estrous cycle, but female *SERT* ^−/−^ mice lacked a normal estrus cycle and appeared to stay in diestrus for up to 14 days (Fig. 3a; Supplementary Table 1). Consistent with a normal estrous cycle, plasma 17β-estradiol levels in female WT mice fluctuated, but the circulating 17β-estradiol in female *SERT* ^−/−^ mice stayed at a lower level (Fig. 3b). We subsequently assessed Cyp19a1 expression in ovaries. Consistent with low circulating levels of 17β-estradiol, ovarian expression of the Cyp19a1 mRNA and protein levels were significantly reduced in female *SERT* ^−/−^ mice compared with WT (Fig. 3c,d). These findings suggest that low 17β-estradiol levels, as a result of Cyp19a1 down regulation, underlie obesity and metabolic abnormalities in female *SERT* ^−/−^ mice. To further test this hypothesis, we implanted slow-release 17β-estradiol or placebo pellets into female WT and *SERT* ^−/−^ mice. Although there was no detectable difference in body mass (Fig. 3e), female *SERT* ^−/−^ mice implanted with 17β-estradiol pellets had a significant reduction of WAT weights (gonadal, inguinal, and retroperitoneal) and an increase in BAT mass compared to those implanted with placebo (Fig. 3f,g). Furthermore, 17β-estradiol treatment improved glucose tolerance and insulin sensitivity, and lowered fed blood glucose levels in female *SERT* ^−/−^ mice (Fig. 3h–j).

Since decreased estrogen levels in males also lead to increased fat mass^29^, we investigated whether loss of SERT in male mice leads to adiposity, glucose intolerance and insulin sensitivity and whether it is associated with a reduction of estrogen concentrations. Indeed, male *SERT* ^−/−^ mice displayed adiposity, glucose intolerance and insulin resistance accompanied with a reduction of circulating 17β-estradiol levels (Supplementary Fig. 2a–e). Furthermore, circulating 17β-estradiol levels inversely correlated with weights of WAT including epididymal (Supplementary Fig. 2f), inguinal (R = −0.642, *P* = 0.007) and retroperitoneal (R = −0.585, *P* = 0.017) depots in male *SERT* ^−/−^ mice. Thus, the reduction of estrogen levels is likely the underlying reason for the development of adiposity, insulin resistance and glucose intolerance in both male and female *SERT* ^−/−^ mice.

### Serotonin reuptake inhibitor treatment also leads to abnormal fat accumulation and insulin resistance in association with estrogen suppression in mice

**Figure 4:**
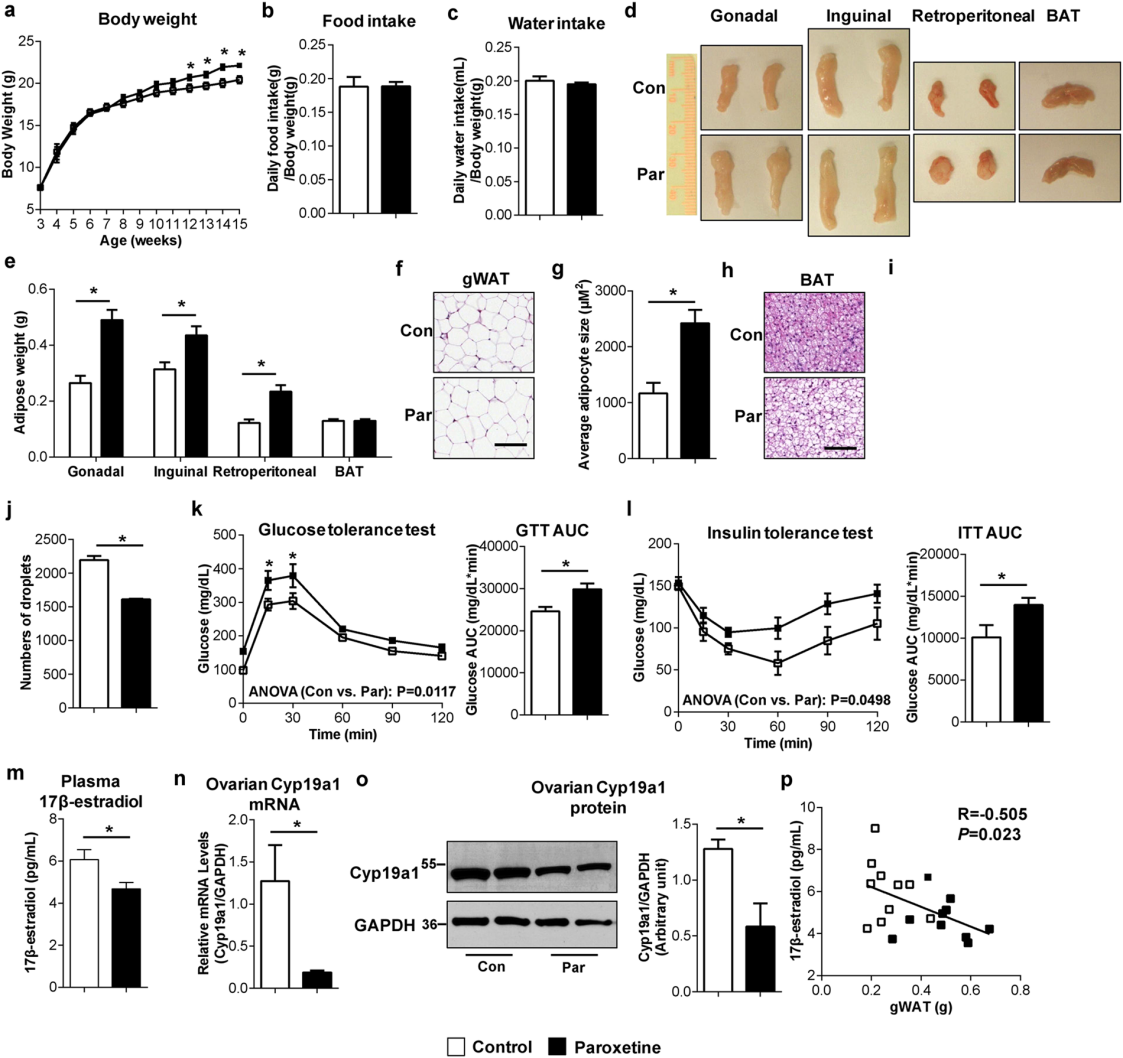
Paroxetine treatment leads to abnormal fat accumulation, insulin resistance and glucose intolerance associated with estrogen suppression in female mice. (**a**–**c**) Body weight, food and water intakes of WT female mice treated with or without paroxetine for 12 weeks (10 mg/kg/day) (n = 10 per group). (**d** and **e**) Representative fat tissues and weights at time of sacrifice (n = 10 per group). (**f**) Representative images of H&E-stained gWAT sections (Scale bars: 100 µm). (**g**) Average adipocyte size per 10× field quantified using ImageJ. (**h**) Representative images of H&E-stained BAT sections (Scale bars: 100 µm). (**i** and **j**) Average lipid droplet area and number per 20× field in BAT was quantified using ImageJ. (**k** and **l**) GTT and ITT were performed after12 weeks of paroxetine treatment in WT female mice (n = 5 per group). The repeated measures ANOVA P value is provided. The corresponding GTT AUC and ITT AUC were calculated. (**m**) Concentrations of 17β-estradiol were measured in plasma from WT female mice treated with or without paroxetine for 12 weeks (n = 5 per group). (**n**) Cyp19a1 mRNA expression in ovary was quantified by RT-qPCR, normalized to GAPDH, and expressed relative to the control group (n = 5 per group). (**o**) Cyp19a1 protein levels in ovary were quantified by western blotting (n = 4 per group). Uncut blots are included in the Supplementary information. (**p**) Spearman rank correlation analysis between plasma 17β-estradiol concentrations and gWAT weights. The Spearman’s rank correlation coefficient and accompanying *P* value are provided. *P < 0.05. Values are reported as mean ± SEM.

To exclude the possibility that the observed estrogen suppression in *SERT* ^−/−^ mice is a congenital effect secondary to *SERT* ^−/−^ deletion, we treated female WT and *SERT* ^−/−^ mice after weaning 3 weeks after birth with paroxetine (an SSRI) in the drinking water for 12 weeks. Body weight, food intake and water consumption were monitored weekly. Compared to vehicle-treated control mice, WT mice treated with paroxetine had no change in daily food and water intake but showed a small (10.8%) increase in total body weight after treatment for 12 weeks (Fig. 4a–c). Similar to female *SERT* ^−/−^ mice, paroxetine-treated female WT mice exhibited higher fat content and their gonadal, inguinal and retroperitoneal WAT depots were significantly larger than those of control mice (Fig. 4d,e). WAT adipocyte cell size was again larger in paroxetine-treated female mice than vehicle controls (Fig. 4f,g). Although BAT depot showed no weight difference (Fig. 4d,e), paroxetine-treated mice appeared to contain a higher amount of unilocular fat droplets in BAT (Fig. 4h–j). Consistent with abnormal fat deposition, paroxetine-treated WT mice exhibited reduced glucose tolerance (Fig. 4k) and insulin sensitivity (Fig. 4l). Importantly, a reduction in plasma 17β-estradiol levels and ovarian Cyp19a1 expression occurred in paroxetine-treated female mice (Fig. 4m–o). Plasma 17β-estradiol levels in individual mice appear to be negatively correlated with weights of WAT including gonadal (Fig. 4p), inguinal (R = −0.453, *P* = 0.045) and retroperitoneal (R = −0.500, P = 0.026) depots. In contrast, paroxetine-treated female *SERT* ^−/−^ mice showed minimal changes in weight gain, fat mass expansion, glucose and insulin tolerance, as well as plasma 17β-estradiol levels (Supplementary Fig. 3), providing a strong evidence that the low plasma 17β-estradiol levels and associated metabolic abnormalities in female mice was a direct consequence of impaired SERT function. The same paroxetine treatment study was also conducted in a cohort of male mice and paroxetine treatment resulted in adipose tissue expansion and glucose intolerance associated with estrogen suppression (Supplementary Fig. 4). Therefore, chronic inhibition of SERT by paroxetine recapitulated the phenotypes of SERT deletion, suppressing estrogen synthesis while inducing abnormal fat deposition, insulin resistance, and glucose intolerance.

### Pregnancy reverses abnormal fat accumulation and glucose intolerance in female *SERT*^−/−^ mice

**Figure 5:**
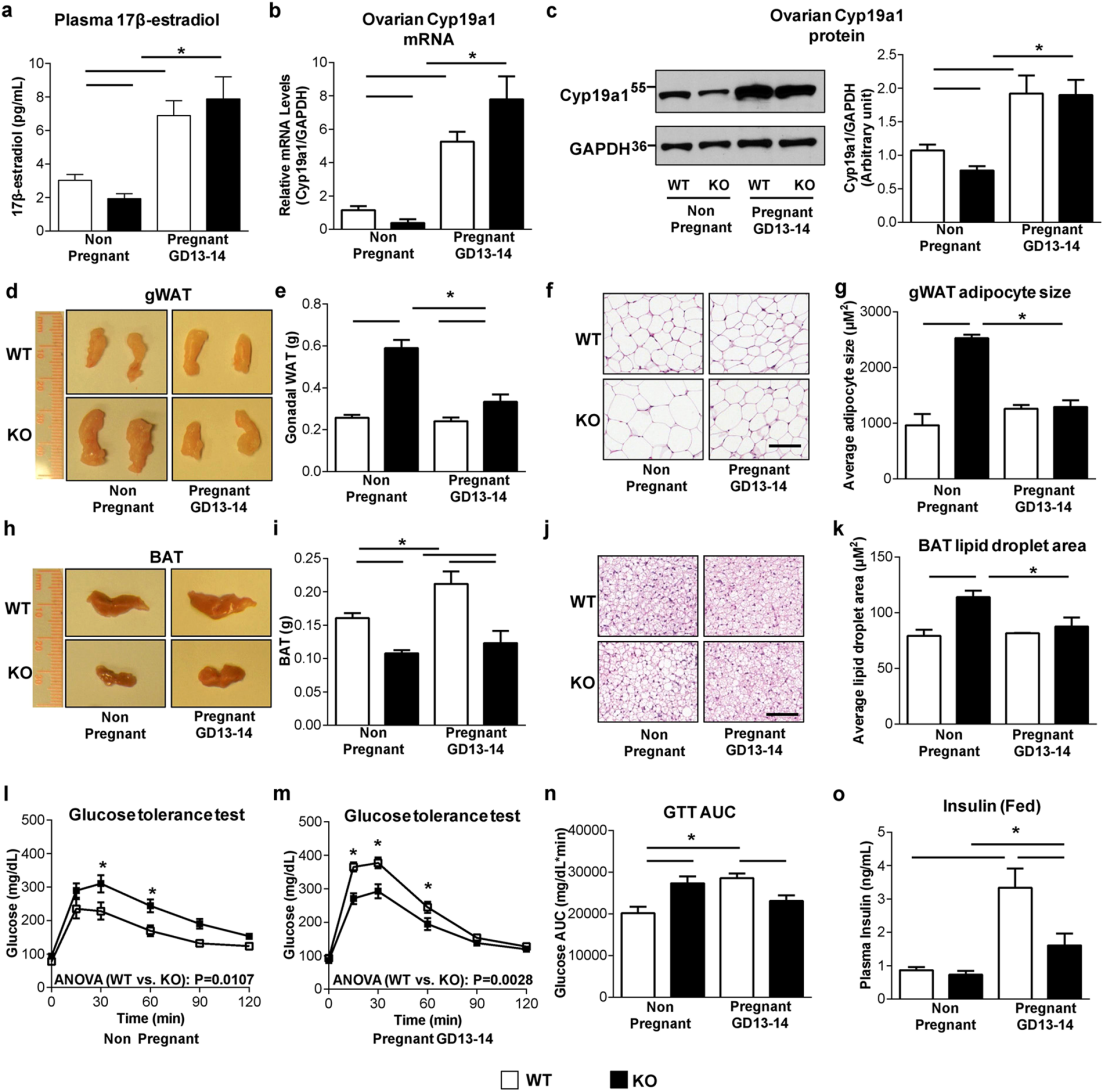
Pregnancy reverses abnormal lipid accumulation and glucose intolerance in *SERT* ^−/−^ mice. (**a–c**) Plasma 17β-estradiol levels (n = 11–13 per group), ovarian Cyp19a1 mRNA (n = 5–6 per group) and protein levels (n = 3 per group) were measured in WT and *SERT* ^−/−^ non-pregnant and pregnant (GD 13–14) mice. Uncut blots are included in the Supplementary information. (**d** and **h**) Representative gWAT and BAT from WT and *SERT* ^−/−^ non-pregnant and pregnant (GD 13–14) mice. (**e** and **i**) gWAT and BAT weight at time of necropsy (n = 6–14 per group). (**f** and **j**) Representative images of H&E-stained gWAT and BAT sections (Scale bars: 100 µm). (**g** and **k**) Average adipocyte size per 10× field in gWAT and average lipid droplet area per 20× field in BAT were quantified using ImageJ. (**l–n**) GTT was performed on WT and *SERT* ^−/−^ non-pregnant and pregnant (GD 13–14) mice (n = 6–11 per group). The repeated measures ANOVA P value is provided. The corresponding GTT AUC was calculated. (**o**) Fed plasma insulin levels were measured (n = 6–7 per group). Studies were performed at 3-month-old mice. *P < 0.05. Values are reported as mean ± SEM.

During pregnancy, estrogen level surges at mid-gestation, which is important for maintaining normal pregnancy^30^. As demonstrated above, estrogen suppression is a major cause of abnormal fat deposition and glucose metabolism dysregulation in non-pregnant female *SERT* ^−/−^ mice. A question arises whether SERT deficiency would affect estrogen surge as well as adipose function and glucose homeostasis during pregnancy. We thus measured 17β-estradiol levels, ovary Cyp19a1 expression, WAT and BAT morphology, and *in vivo* glucose tolerance in 3-month old pregnant WT and *SERT* ^−/−^ mice at gestational day (GD) 13–14. As illustrated in Fig. 5a, plasma concentrations of 17β-estradiol in 3-month old pregnant WT and *SERT* ^−/−^ mice increased significantly to a similar level at mid-gestation. Concurrently, ovarian Cyp19a1 mRNA and protein expression increased substantially in both WT and *SERT* ^−/−^ mice (Fig. 5b,c). Along with the elevated circulating estrogen levels in pregnant mice, the increase in the weights of gWAT and adipocytes size seen in non-pregnant *SERT* ^−/−^ mice was almost completely blunted in pregnant *SERT* ^−/−^ mice (Fig. 5d–g). BAT mass was increased by pregnancy in both WT and *SERT* ^−/−^ mice (Fig. 5h,i), although pregnant *SERT* ^−/−^ mice still had significantly lower BAT mass compared to WT mice. In addition, lipid droplet area was significantly reduced in pregnant *SERT* ^−/−^ mice leading to no observable difference between pregnant WT and *SERT* ^−/−^ mice (Fig. 5j,k). Consistent with less adiposity, a reversal of glucose tolerance was observed in pregnant *SERT* ^−/−^ mice which showed even better glucose control than WT pregnant controls (Fig. 5l–n). This reversal in glucose homeostasis was also observed in another cohort of 6-month old female WT and *SERT* ^−/−^ mice (Supplementary Fig. 5). Plasma insulin levels were significantly lower in pregnant *SERT* ^−/−^ mice compared to pregnant WT controls (Fig. 5o), suggesting that the improved glucose control was not due to an increase in insulin production, but rather a better insulin action in pregnant *SERT* ^−/−^ mice. This interpretation is further supported by a reduction of pancreatic insulin content (Supplementary Fig. 6a) and a trend of reduction in pancreas weight (Supplementary Fig. 6b) and β cell mass (Supplementary Fig. 6c,d) in pregnant *SERT* ^−/−^ mice compared to pregnant WT controls. Collectively, our findings demonstrate that estrogen suppression due to SERT impairment is restored during pregnancy through increased 17β-estradiol synthesis by a SERT-independent mechanism, resulting in an improved fat deposition and glucose control in pregnant *SERT* ^−/−^ mice.

### ERα is necessary for pregnancy-induced improvements in adipose tissue and glucose homeostasis in *SERT*^−/−^ mice

**Figure 6:**
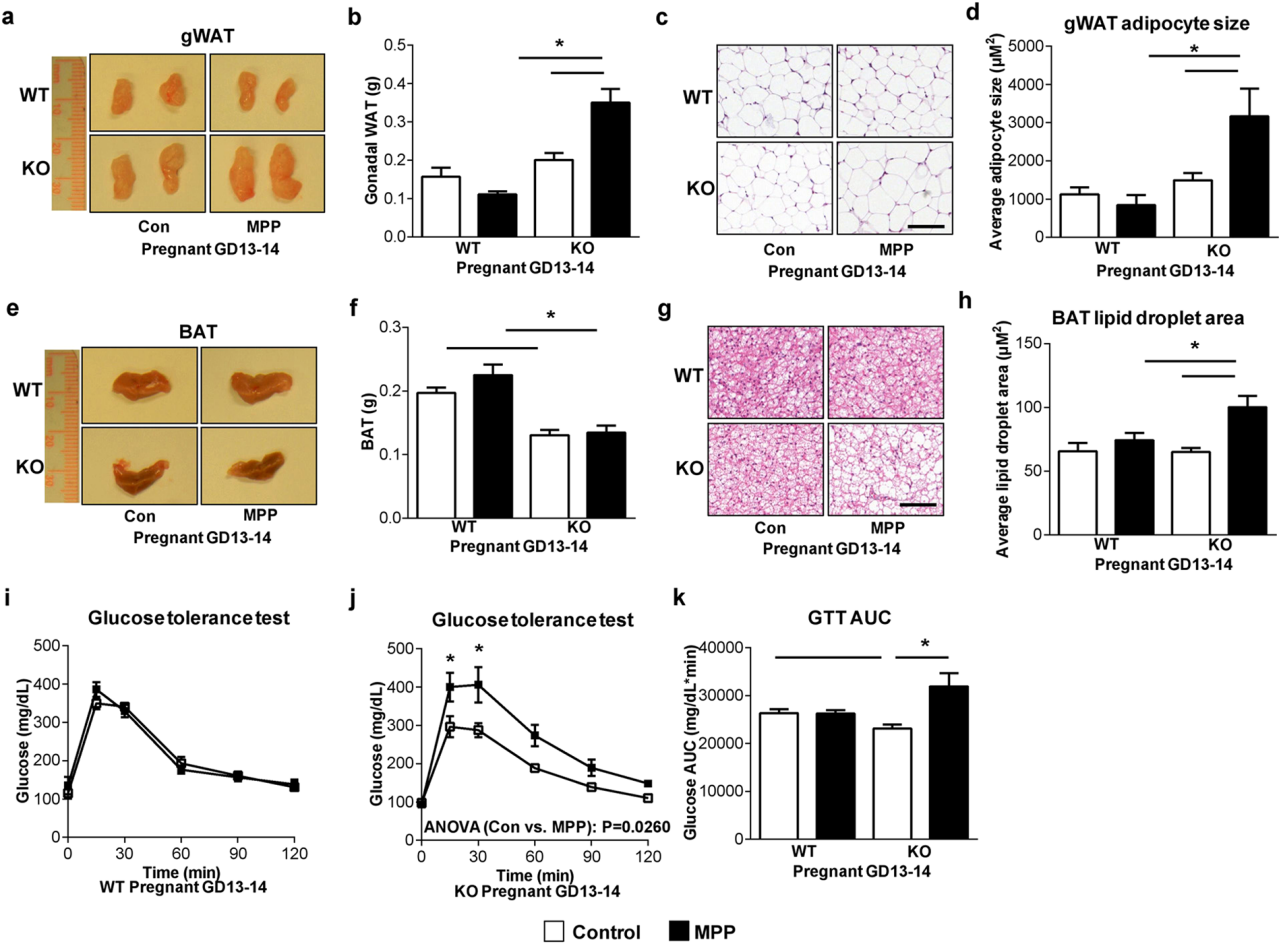
ERα signaling is required for the reverse of adiposity and glucose intolerance by pregnancy in *SERT* ^−/−^ mice. (**a** and **e**) Images of representative gWAT and BAT from WT and *SERT* ^−/−^ pregnant (GD 13–14) mice treated with MPP (i.p. 0.5 mg/kg/day) or vehicle from day 7 to day 13–14 of pregnancy (n = 5–6 per group). (**b** and **f**) gWAT and BAT weight at time of necropsy (n = 5–6 per group). (**c** and **g**) Representative images of H&E-stained gWAT and BAT sections (Scale bars: 100 µm). (**d** and **h**) Average adipocyte size per 10× field in WAT and average lipid droplet area per 20× field in BAT were quantified using ImageJ. (**i**-**k**) GTT was performed in WT and *SERT* ^−/−^ pregnant (GD 13–14) mice treated with or without MPP (n = 5–6 per group). The repeated measures ANOVA P value is provided. The corresponding GTT AUC was calculated. *P < 0.05. Values are reported as mean ± SEM.

The estrogen/ERα signaling has been shown to be associated with obesity and the development of adipose tissue^31^. Independent of gender, mice lacking ERα have an increase in fat pad weights, insulin resistance and impaired glucose tolerance^31, 32^. In fact, we had found ERα mRNA levels were higher in gonadal fat of pregnant *SERT* ^−/−^ mice (Supplementary Fig. 7). Recent studies have shown that the small molecule ERα antagonist methyl-piperidino-pyrazole (MPP) could effectively block ERα activation *in vivo* ^33–35^. Therefore, to assess the contribution of ERα signaling in the improvement of adiposity in pregnant *SERT* ^−/−^ mice, we treated pregnant WT and *SERT* ^−/−^ mice with MPP from GD 7 to GD 13–14. Pregnant *SERT* ^−/−^ mice receiving MPP exhibited increased gWAT (Fig. 6a,b), as well as white adipocyte cell size (Fig. 6c,d). Although there was no effect on BAT mass (Fig. 6e,f), MPP administration led to an increase in lipid droplet size and a decrease in multilocular adipocytes in pregnant *SERT* ^−/−^ mice but not in WT mice (Fig. 6g,h). Furthermore, we found that injection of MPP significantly reduced glucose tolerance in pregnant *SERT* ^−/−^ mice but not in WT mice (Fig. 6i–k). Thus, our data suggest that the protective effect of pregnancy on adiposity and glucose tolerance requires ERα signaling in female *SERT* ^−/−^ mice.

## Discussion

**Figure 7:**
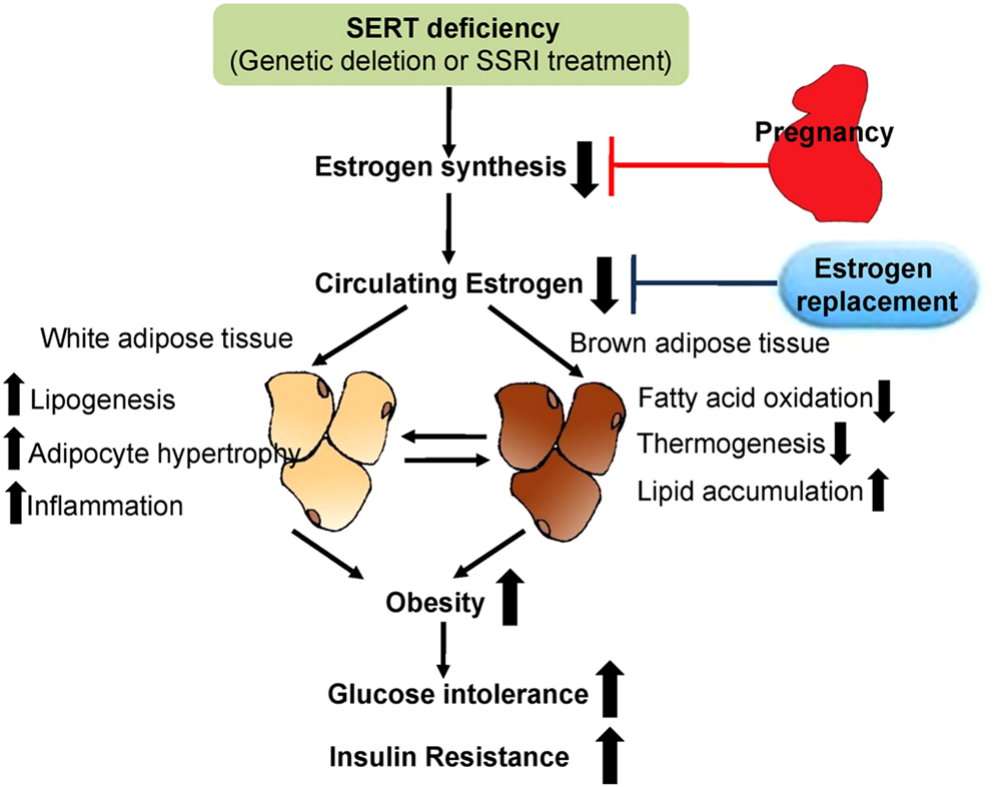
SERT deficiency leads to adiposity and abnormal glucose metabolism through estrogen suppression *in vivo*. Genetic deletion or pharmacological inhibition of SERT in mice reduces gonadal estrogen synthesis and plasma 17β-estradiol levels, which lead to the abnormal fat accumulation in both white and brown adipose tissues, further contributing to adiposity and dysregulation of glucose homeostasis at the whole-body level. Restoring estrogen by exogenous estrogen therapy or during pregnancy rescues SERT deficiency-induced adiposity and glucose intolerance.

SERT plays an important role both in depression and its pharmacological treatment. Although SERT deficiency, either by genetic deletion or pharmacological inhibition is associated with obesity and associated metabolic diseases in human and animal models^8–10, 36^, the molecular factors mediating SERT deficiency-associated obesity and related metabolic disorder remains poorly understood. In the present investigation, we provide three lines of evidence to show that SERT deficiency leads to adiposity and abnormal glucose metabolism through estrogen suppression *in vivo* (Fig. 7). The first is that genetic deletion of SERT in mice led to increased visceral adiposity and brown fat lipoatrophy, glucose intolerance and insulin resistance, associated with a reduction of circulating estrogen levels. These metabolic deficits of *SERT* ^−/−^ mice are reversible and can be restored by estrogen replacement. Second, pharmacological blockade of SERT in mice also induces visceral adiposity, impaired glucose and insulin tolerance in association with reduced circulating 17β-estradiol levels. Third, pregnancy along with increased circulating estrogen levels significantly improves abnormal fat deposition and glucose metabolism in pregnant *SERT* ^−/−^ mice via ERα signaling. Taken together, these findings demonstrate that estrogen suppression provides a novel biological link between SERT deficiency and development of abnormal fat deposition and glucose metabolism.

In humans, obesity is significantly associated with a polymorphism of low SERT expression or chronic use of SSRIs^9, 37–39^. Women who take SSRIs are reportedly to gain a larger amount of weight compared to male counterparts^40, 41^. However, the impact of SERT deficiency on adipocyte morphology and function in both genders has never been investigated. Our analyses of adipose tissues from female and male *SERT* ^−/−^ mice revealed markedly expanded WAT mass and adipocyte size as well as reduced BAT mass and abnormal lipid accumulation in BAT. These changes are accompanied by significantly altered expression of genes involved in lipid synthesis (PPARγ, SREBP1c, Fabp4, and LPL), triglyceride breakdown (HSL and ATGL), thermogenesis (UCP1) and oxidative response (PGC1α, PPARα, and CPT1b). WT mice under chronic paroxetine treatment also showed significant WAT expansion and abnormal lipid accumulation in BAT at a dose comparable to the therapeutic levels in humans^42^. These findings suggest that SERT deficiency induces adiposity and abnormal lipid accumulation in both WAT and BAT, leading to insulin resistance and glucose intolerance in both female and male mice.

A remarkable finding of our current study was the involvement of estrogen reduction in SERT deficiency-associated adiposity and dysregulation of glucose homeostasis. We first found that SERT deficiency impairs the estrous cycle and reduces gonadal estrogen synthesis and circulating estrogen levels. It is well-established that estrogen reduction promotes adiposity and insulin resistance in both rodents and humans^18, 21, 43^, together with our results presented here, led us to postulate that circulating estrogen reduction contributes to the development of adiposity and glucose intolerance in SERT deficiency mice. Indeed, we found reduced Cyp19a1 expression and decreased circulating 17β-estradiol levels, which are inversely correlated with adipose tissue weights and glucose tolerance in *SERT* ^−/−^ mice and paroxetine-treated WT mice. Moreover, 17β-estradiol replacement in female *SERT* ^−/−^ mice markedly reduced fat mass, improved glucose tolerance and insulin sensitivity, further supporting that estrogen suppression is responsible for adiposity and abnormal glucose metabolism in these animals. While we have not been able to directly investigate if estrogen plays a role in SERT deficiency related obesity and diabetes in humans, several lines of evidence suggest that a similar mechanism may also be involved. First, while estrogens are synthesized in both men and women, they are present at significantly higher levels in women of reproductive age. Interestingly, women who take SSRIs reportedly gain a larger percent of weight compared to men^40^. This could be due to a larger role of estrogen in regulating adipose distribution in women. Second, in a small cohort of 35 healthy males, treatment with paroxetine was reported to affect sperm DNA fragmentation along with a significant reduction of serum 17β-estradiol levels^44^. Lastly, estrogen also plays other important roles such as promoting bone health and sexual drive. Loss of libido is a common side effect associated with SSRI use^45^ and SSRI use is repeatedly reported as a risk factor for treatment-related bone loss and fracture^46, 47^. Thus, estrogen suppression may also be involved in other adverse effects of SSRIs. Further clinical research is necessary to confirm such a connection that may help to explain and manage the adverse effects caused by long-term use of SERT inhibitors.

It has been well documented that normal pregnancy reduces insulin sensitivity in its target tissues and compensatory increases insulin production to maintain the normal glucose levels both in human and animal models^48–50^. Indeed, we found reduced insulin sensitivity and increased insulin production in WT pregnant female mice, in agreement with previous reports^51^. These mice had normal blood glucose levels compared to non-pregnant controls. However, we observed glucose intolerance was significantly improved in female *SERT* ^−/−^ mice without changes in insulin levels and β-cells function, suggesting that pregnancy improves insulin sensitivity in female *SERT* ^−/−^ mice. The opposite effect of pregnancy on insulin sensitivity led us to speculate that there is a unique mechanism of induction of insulin action in pregnant *SERT* ^−/−^ mice. Indeed, our findings reveal that pregnancy specifically reduce lipid accumulation in WAT and BAT in female *SERT* ^−/−^ mice, as abnormal lipid accumulation in WAT and BAT plays a key role in the pathogenesis of insulin resistance^52, 53^. Moreover, this effect over whole-body metabolism is through an estrogen/ERα signaling-dependent mechanism, as pregnant *SERT* ^−/−^ mice treated with an ERα antagonist, MPP, lost the improvement in adiposity and glucose intolerance compared to WT pregnant female mice. ERα-deficient mice have increased WAT mass and impaired glucose tolerance^31^, similar to the pregnant *SERT* ^−/−^ mice in the current study that were treated with MPP. Our studies also demonstrate an increased lipid accumulation in BAT of *SERT* ^−/−^ pregnant female mice treated by ERα antagonist MPP, suggesting that pregnancy improves BAT function in pregnant *SERT* ^−/−^ mice through ERα signaling. However, the estrogen effects on BAT function may be centrally or directly mediated^54^. One recent study indicates that 17β-estradiol could increase BAT thermogenesis via central ERα signaling^55^, suggesting that there is a possibility that pregnancy increases BAT thermogenesis via a central ERα signaling pathway. Nevertheless, further investigation is needed on this aspect.

There are a few limitations of our studies. First, while we showed that the reduced circulating 17β-estradiol levels in *SERT* ^−/−^ and SSRI-treated mice were associated with a reduction in Cyp19a1 expression; potential changes in estrogen metabolism were not analyzed. As an increase in estrogen catabolism may also contribute to decreased circulating 17β-estradiol levels, future studies are needed to investigate if estrogen-metabolizing enzymes might also be affected by SERT deficiency. Second, how SERT deficiency suppresses Cyp19a1 expression in non-pregnant state and how this suppression is eradicated by pregnancy remain to be established. In most mammals, the production of estrogen in gonads is under the control of the hypothalamic-pituitary-gonadal (HPG) axis through the coordinated action of gonadotropin-releasing hormone and gonadotropins. In females, the synchronized expression of the CYP19 gene in the ovary plays a key role in the normal progression of the menstrual/estrous cycle. However, during pregnancy, the HPG axis is suppressed and estrogen is mainly produced by the placenta in humans due to a marked induction of placental CYP19 in mid gestation^56^. Although rodents do not express Cyp19a1 in placenta, the drastic increase in ovarian Cyp19a1 expression at mid-gestation is directly regulated by placental lactogens^57^. Thus, SERT deficiency appears to only affect Cyp19a1 regulatory pathways in the non-pregnant state. The elevated brain extracellular 5-HT levels in the *SERT* ^−/−^ or SSRI-treated mice may disrupt the normal function of the HPG axis, leading to a dysregulated estrogen synthesis in the ovary in the non-pregnant state. Additionally, 5-HT may regulate gonadal expression of Cyp19a1 in a direct manner, as a functional system for serotonin synthesis, reuptake and receptor activation has been identified in female reproductive tissue^58^. Future studies are needed to delineate the key signaling pathways that link SERT deficiency and reduced Cyp19a1 expression.

In summary, our findings indicate that genetic deficiency of SERT as well as inhibition of SERT by SSRI lead to abnormal fat deposition in WAT and BAT, glucose intolerance and insulin resistance *in vivo*. These effects are to be due to suppressed aromatase expression and reduced circulating estrogen levels in SERT deficient mice. Even more, estrogen replacement results in an improvement in adiposity and glucose metabolism. Our studies further demonstrate that pregnancy has beneficial effects on the control of fat composition in WAT and BAT and glucose metabolism in SERT deficient female mice through a normalized circulating estrogen level and activation of ERα signaling. This work demonstrated a direct effect of SERT on estrogen synthesis and further identified estrogen as a critical factor that contributes to depression treatment-induced obesity and metabolic abnormalities.

## Methods

### Mouse studies

All mice used in the experiments were bred on a C57BL/6 background and housed under standard conditions (12:12-h light-dark cycle; water and food ad libitum) in specific pathogen-free barrier facilities. The institutional animal care and use committee at the University of Washington approved all of the procedures involving mice and the experiments were carried out in accordance with the approved guidelines and regulations. The breeding pairs of WT and *SERT* ^−/−^ mice^59^ were obtained from the Jackson Laboratory (Bar Harbor, ME). *SERT* ^−/−^ mice were generated by breeding heterozygous females with either heterozygous or homozygous male *SERT* ^−/−^ mice, and all genotypes were confirmed by PCR. In all experiments, genotype-, gender- and age-matched mice were randomized into different study groups. For the SSRI studies, female and male WT and *SERT* ^−/−^ mice were treated with paroxetine hydrochloride (10 mg/kg/day; Ark Pharma, Inc, Libertyville, IL) in drinking water from weaning at 3 week-old for 12 weeks. Body weight, food intake and water consumption were monitored weekly. For the 17β-estradiol replacement study, a single placebo or 17β-estradiol-containing pellet delivering 5 µg/day of hormone (Innovative Research of America) was implanted subcutaneously in 8-week-old female *SERT* ^−/−^ mice. Mice were treated and monitored for a total of 3 weeks and were subjected to GTT and ITT tests before euthanasia. For pregnant studies, female WT and *SERT* ^−/−^ mice at GD 13–14 were used. Successful mating was confirmed by the detection of a vaginal plug the next morning, which was assigned as day 0 of gestation (GD 0)^60^. Body weight and food intake were monitored daily from GD 0 to GD 13–14. For the ERα antagonist treatment study in pregnant mice, pregnant WT and *SERT* ^−/−^ mice (age 8–12 weeks) received either MPP dihydrochloride (0.5 mg/kg/day; Sigma-Aldrich, St. Louis, MO) or vehicle (10% ethanol) once daily via intraperitoneal injection from day 7 to day 13–14 of pregnancy. At the end of the experiments, mice were euthanized by CO_2_ inhalation. Blood was collected via cardiac puncture, and plasma was separated by centrifugation. Various fat pads (gonadal, epididymal, inguinal, retroperitoneal, and brown adipose tissues), pancreas, and ovary were harvested and weighed; one part of the tissue was snap-frozen in liquid N_2_ and stored at −80 °C, while the remainder was fixed in 10% formalin and embedded in paraffin for histological analysis.

### Adipose histology

WAT and BAT were processed to paraffin-embedded sections (5 μm sections, 100 μm apart), and then were stained with H&E and scanned into digital images (ScanScope CS; Aperio, Vista, CA). WAT adipocyte size per 10× field and BAT lipid droplets size and number per 20× field were quantified using ImageJ software (NIH) and the MRI adipocyte tool, as described^61, 62^. An average value across nine non-overlapping fields (three fields/section × three sections/mouse) was calculated for each mouse.

### RNA isolation and quantitative PCR

Total RNA was extract from WAT, BAT, and ovary using the RNeasy Miniprep kit (Qiagen, Valencia, CA). Total RNA (2 μg) was reverse transcribed to cDNA, as described^60, 63^. Expression of murine *PPARγ* (Mm01184322_m1), *SREBP1c* (Mm00550338_m1), fatty acid binding protein 4 (*Fabp4*) (Mm00445878_m1), *LPL* (Mm00434764_m1), *PPARα* (Mm00440939_m1), *CPT1b* (Mm00487200_m1), *HSL* (Mm00495359_m1), *ATGL* (Mm00503040_m1), *TNF-α* (Mm00443258_m1), *IL-6* (Mm00446190_m1), *PGC1α*(Mm01208835_m1), uncoupling protein 1 (*UCP1*) (Mm01244861_m1), *Cyp19a1* (Mm00484049_m1), *Esr1* (Mm00433149_m1) and *GAPDH* (Mm99999915_g1) were quantified using TaqMan® Assays on Demand (Life technologies) as described previously^64^. Gene expression was normalized to GAPDH expressed relative to control using the 2^−ΔΔCt^ method^65^.

### Glucose and insulin tolerance tests

Glucose tolerance test (2 g/kg body weight, injected intraperitoneally) was performed in male, female, and pregnant (GD 13–14) mice after a 16-h overnight fast. Insulin tolerance test (0.75 units insulin/kg body weight, injected intraperitoneally, Humulin R, Lilly) was performed after a 6 h fast. Whole blood glucose concentrations were measured before and 15, 30, 60, 90, and 120 min following dosing with a One-Touch Ultra Glucometer (LifeScan, Milpitas, CA). The area under the glucose concentration-time curve (glucose AUC_0–120min_) was calculated using the trapezoidal method.

### Glucose uptake ***in vivo***

Glucose uptake *in vivo* using a non-metabolizable glucose analog [^3^H]2-deoxyglucose ([^3^H]2-DG) was measured as described^28^. Briefly, mice were fasted 16 h and then dosed i.p. with [^3^H]2-DG (60 mCi/mmol; 400 μCi/kg body weight) and glucose (2 g/kg body weight). Blood samples were obtained as described above for glucose tolerance test. Radioactivity was determined by a liquid scintillation counter. After 120 min following injection, the mice were euthanized and gWAT, liver, and skeletal muscle were collected. The gWAT and skeletal muscle were processed to determine their [^3^H]2-DG-6P concentration, while liver was processed to determine glycogen concentration. As 2-DG-6P is not the main product of 2-DG in liver, the incorporation of radioactivity into glycogen was used as an index of 2-DG uptake in liver^66^.

Tissue glucose uptake (Rg) was calculated using the equation:

$${\rm{Rg}}=[{}^{3}{\rm{H}}]2-{\rm{DG}}-6{{\rm{P}}}_{{\rm{tissue}}}({\rm{or}}[{}^{3}{\rm{H}}]{{\rm{Glycogen}}}_{{\rm{liver}}})\times {{\rm{Glucose}}}_{{\rm{plasma}}}/{\rm{AUC}}[{}^{3}{\rm{H}}]2-{{\rm{DG}}}_{{\rm{plasma}}}$$

where Glucose_plasma_ was the average plasma glucose concentration over the 120 min of the experiment and AUC[^3^H]2-DG plasma is the area under the curve for plasma radio activity^67^.

### Immunoblotting

Protein was extracted from mouse tissues using RIPA buffer containing 50 mM Tris-HCl (pH 8.0), 1% Triton X 100, 150 mM NaCl, 1 mM EDTA, 0.25% sodium deoxycholate, 1 mM sodium fluoride, 1 mM sodium or thovanadate and protease inhibitor mixture (Roche Applied Science). Protein concentrations were determined with a Pierce BCA Protein Assay Kit (Thermo Scientific). Protein samples were subjected to SDS-PAGE, and then transferred to polyvinylidene fluoride (PVDF) membrane (Millipore Corporation). For ovarian Cyp19a1 expression determination, the blot was incubated with an anti-Cyp19a1 polyclonal antibody (1:500, Santa Cruz Biotechnology) and followed by detection with a horseradish peroxidase-conjugated mouse anti-goat IgG (1:4,000, Santa Cruz Biotechnology). For insulin signaling experiments, the blot was incubated with phospho-Akt (Ser473) or an Akt antibody (1:1000, Cell Signaling) and followed by detection with a horseradish peroxidase-conjugated goat anti-rabbit IgG (1:4,000, Santa Cruz Biotechnology). Immunoreactive bands were detected by chemiluminescence using the ECL Western blotting substrate (Thermo Scientific). The density of the immunoreactive bands was analyzed using ImageJ software (NIH). To measure insulin-induced Akt activation, after an overnight fast, mice were injected (i.p.) with insulin (0.75 unit/kg body weight), white adipose tissue and liver were collected 10 min after injection, and western blot analyses were performed.

### Determination of estrous stage

The stage of the estrous cycle was determined by vaginal lavage and cytological analysis that have been previously described^68, 69^. Smears were classified as diestrus, proestrus, estrus and metestrus based on microscopic examination of vaginal epithelial cell morphology. All mice were evaluated for 20 days in which the first 6 days were excluded to allow the mice to acclimatize to the procedure.

### ELISA

Plasma 17β-estradiol levels were determined using a commercial mouse ELISA kit (CalBiotech). Plasma insulin levels were measured using a mouse ELISA kit (Mercodia).

### Statistical analysis

All data are reported as mean ± SEM. When data was normally distributed, a 2-tailed Student’s t test was employed to analyze the difference across two groups, and one-way ANOVA with a post hoc Dunnett’s test was used to determine the difference between multiple experimental groups. One-way ANOVA with a post hoc Wilcoxon rank-sum test was used for data that were not normally distributed. Differences in the glucose concentration-time profile were determined using two-way repeated-measures ANOVA and Bonferroni post-hoc tests. Correlation analysis was performed using the Spearman rank correlation. Statistical analysis was performed using GraphPad Prism 6 (GraphPad Software, Inc., San Diego, CA), and *P* < 0.05 was considered significant.

## Electronic supplementary material

Supplementary Information
