# Supplementary material for: Serotonin transporter deficiency drives estrogen-dependent obesity and glucose intolerance
Source: Sci Rep. 2017 Apr 25;7:1137. doi: 10.1038/s41598-017-01291-5 (PMC5430688; doi:10.1038/s41598-017-01291-5)

Serotonin transporter deficiency drives estrogen-dependent obesity and glucose intolerance

Weibin Zha,[1] Horace T B Ho,[1] Tao Hu,[1] Mary F. Hebert,[2,3] and Joanne Wang[1,4]

[1]Department of Pharmaceutics, University of Washington, Seattle, Washington, USA

[2]Department of Pharmacy, University of Washington, Seattle, WA, USA

[3]Department of Obstetrics & Gynecology, University of Washington, Seattle, WA, USA

[4]Nutrition Obesity Research Center, University of Washington, Seattle, WA, USA

To whom correspondence should be addressed: Joanne Wang, Department of Pharmaceutics, University of Washington, H272J, Health Sciences Bldg., Seattle, WA 98195-7610. Tel.: 206-221-6561; Fax: 206-543-3204; E-mail: jowang@u.washington.edu.

Supplementary Experimental Procedures

Immunohistochemistry and quantification

Pancreatic tissues were collected from each animal and embedded in OCT compound. Sections (5 μm) were prepared from each sample and stained for insulin using guinea pig anti insulin antibodies (Abcam). Detection of the first antibody (1:100) was performed using Alexa Fluor 555 goat anti guinea pig secondary antibodies (Life technologies, 1:500). Nuclei were stained using ProLong® Gold Antifade Reagent with DAPI (Life Technologies). Digital images were captured using a fluorescence microscope (Nikon Ti-S microscope, Nikon Instruments, Inc.) at 10X magnification. Then, the area of islets (more than 5 insulin-positive cells) was quantified using ImageJ software (NIH). The total islet area (insulin-positive area) was divided by the total pancreas area (nuclei-positive area) to obtain the percentage of the beta cell area in each pancreatic section. The β-cell mass was calculated by the % area of β-cells multiplying by the pancreas weight[1].

ELISA

For determination of mouse pancreatic insulin content, pancreatic tissue was homogenized in acid ethanol (0.18 M HCl in 70% ethanol)[1]. The supernatant was collected after centrifugation, and insulin content was measured using a mouse insulin ELISA kit (Mercodia). Pancreatic insulin concentrations were normalized to mg of pancreatic weight.

References

1. Iglesias J, *et al.* PPARbeta/delta affects pancreatic beta cell mass and insulin secretion in mice. *J Clin Invest* **122**, 4105-4117 (2012).

Supplementary Figures

Supplementary Figure 1. SERT deficiency leads to increased visceral adiposity and brown fat lipoatrophy in female mice at 6-month old. (**a**) Body weight of WT and *SERT*$^{-/-}$ female mice at 6-month old (n = 5 per group). (**b** and **c**) Representative fat tissues and weight at time of sacrifice (n = 6-12 per group). (**d**) Representative images of H&E-stained gonadal white adipose tissue (gWAT) sections (Scale bars: 100μm). (**e**) Average adipocyte size per 10× field quantified using ImageJ. (**f**) Representative images of H&E-stained BAT sections (Scale bars: 100 μm). (**g** and **h**) Average lipid droplet area and number per 20× field in BAT was quantified using ImageJ. *P< 0.05. Values are reported as mean ± SEM.

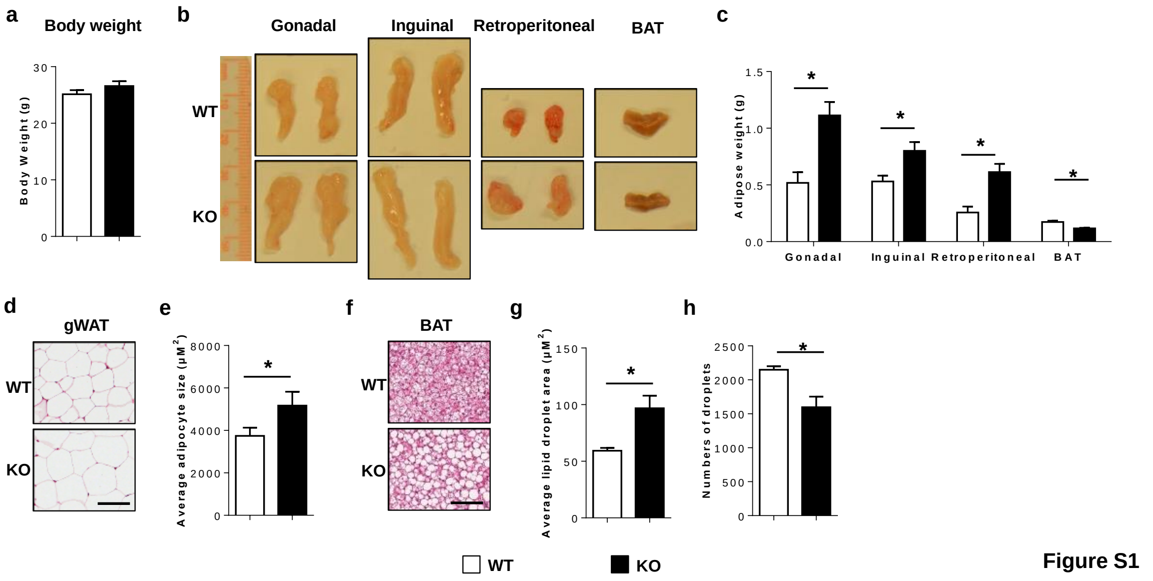

Figure S1

Supplementary Figure 2. SERT deficiency leads to adiposity and glucose intolerance in association with estrogen suppression in male mice. (**a** and **b**) Representative fat tissues and weight of 3-month old WT and *SERT*$^{-/-}$ male mice at time of sacrifice (n = 7-9 per group). (**c** and **d**) Glucose tolerance test (GTT)and insulin tolerance test (ITT) were performed on male WT and *SERT*$^{-/-}$ mice at 3-month old (n = 7-9 per group). The repeated measures ANOVA P value is provided. The corresponding GTT AUC and ITT AUC were calculated. (**e**) Concentrations of 17β-estradiolin plasma were measured from male WT and *SERT*$^{-/-}$ mice(n= 7-9 per group). (**f**) Plasma 17β-estradiol concentration exhibited a significant inverse correlation with epididymal WAT (eWAT) weights. The Spearman's rank correlation coefficient and accompanying *P* value are provided. **P*< 0.05. Values are reported as mean ± SEM.

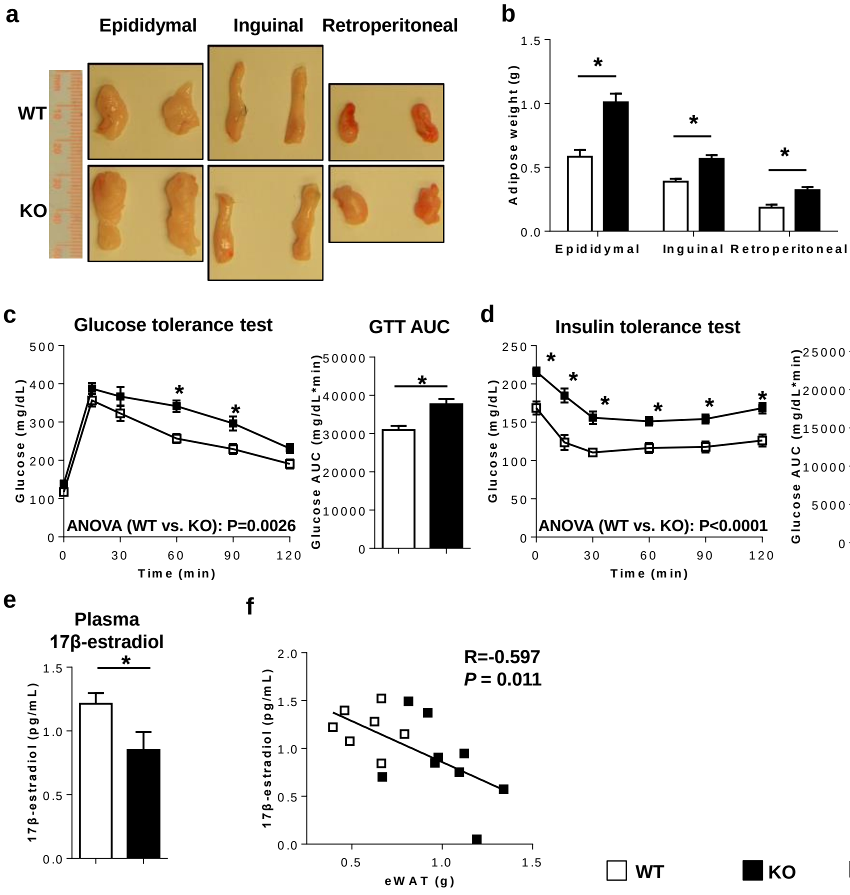

Figure S2

Supplementary Figure 3. Serotonin reuptake inhibitor does not affect adiposity and glucose tolerance in female *SERT*$^{-/-}$ mice. (**a**) Body weight changes of female *SERT*$^{-/-}$ mice treated with or without paroxetine for 12 weeks (10 mg/kg/day) (n = 4 per group). (**b** and **c**) Representative fat tissues and weight at time of sacrifice (n = 4 per group). (**d** and **e**) GTT and ITT were performed after12 weeks of paroxetine treatment in female *SERT*$^{-/-}$ mice (n = 4 per group). The repeated measures ANOVA P value is provided. The corresponding GTT AUC and ITT AUC were calculated. (**f**) Plasma 17β-estradiol levels were measured in female *SERT*$^{-/-}$ mice treated with or without paroxetine for 12 weeks (n=4 per group). **P*< 0.05. Values are reported as mean ± SEM.

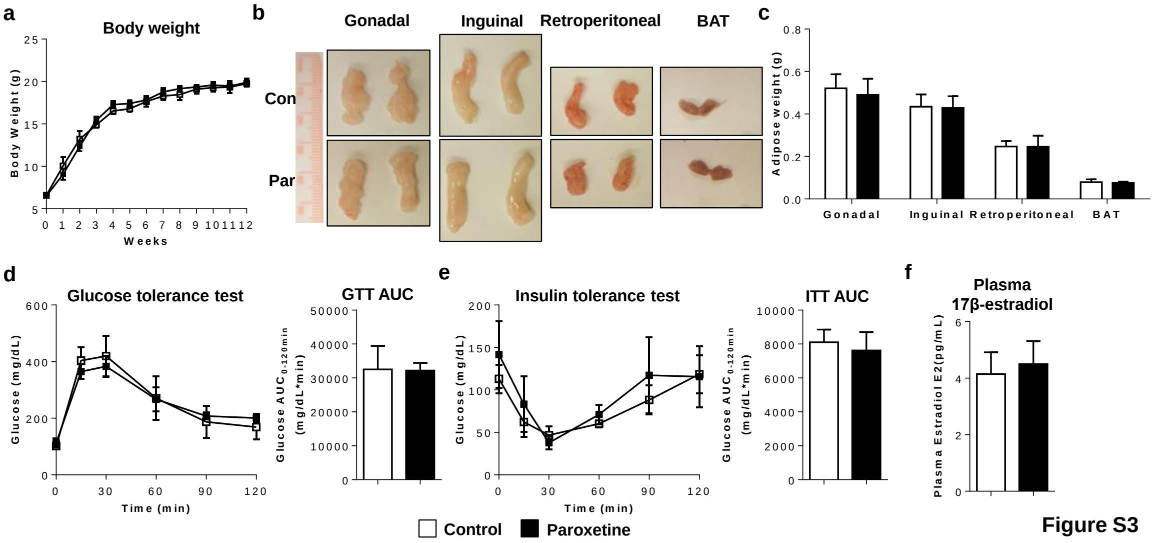

Figure S3

Supplementary Figure 4. Serotonin reuptake inhibitor treatment leads to adiposity and glucose intolerance in association with estrogen suppression in male mice. (**a** and **b**) Representative fat tissues and weight of male WT mice treated with or without paroxetine for 12 weeks (10 mg/kg/day) (n = 5 per group). (**c** and **d**) GTT and ITT were performed after12 weeks of paroxetine treatment in male WT mice. The repeated measures ANOVA P value is provided. The corresponding GTT AUC and ITT AUC were calculated. (**e**) Concentrations of 17β-estradiol in plasma were measured from male WT mice treated with or without paroxetine for 12 weeks (n = 5 per group). (**f**) Plasma 17β-estradiol concentration exhibited a significant inverse correlation with epididymal WAT (eWAT) weights. The Spearman's rank correlation coefficient and accompanying *P* value are provided. *$P< 0.05$. Values are reported as mean ± SEM.

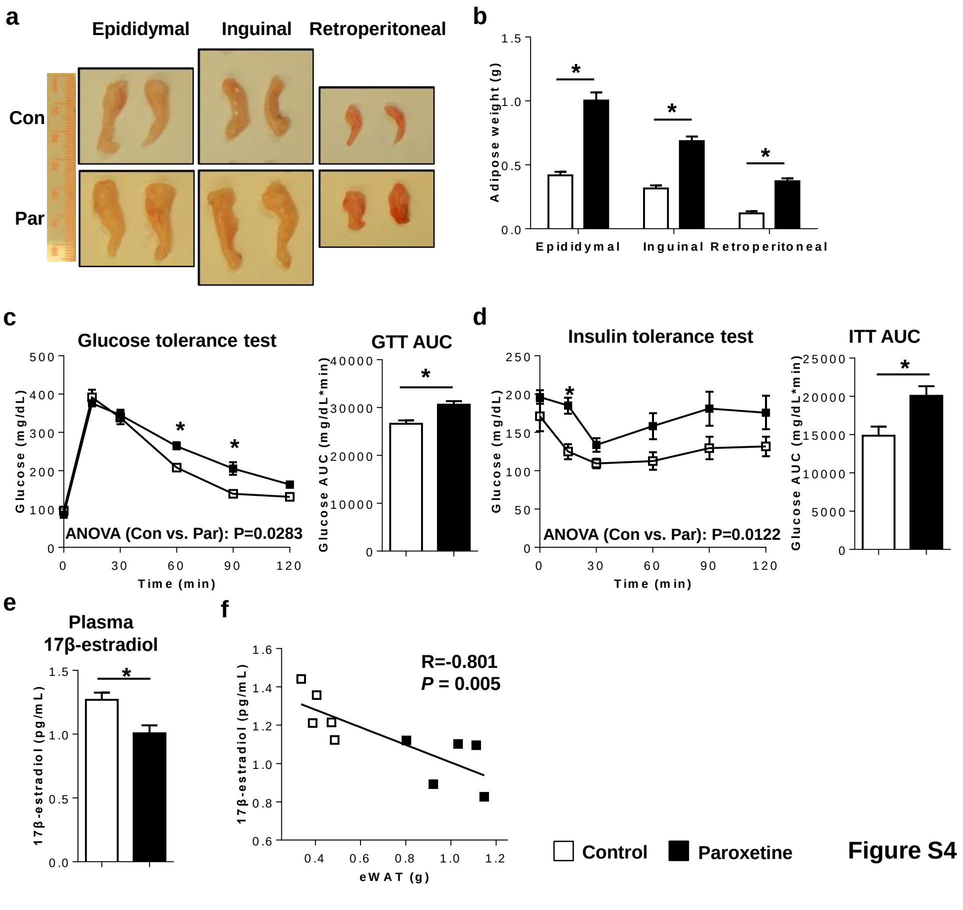

Figure S4

Supplementary Figure 5. Pregnancy reverses glucose intolerance in *SERT*$^{-/-}$ mice at 6-month old. (**a-c**) GTT was performed on non-pregnant and pregnant (GD13-14) WT and *SERT*$^{-/-}$ mice at 6-month old (n = 7-8 per group). The repeated measures ANOVA P value is provided. The corresponding GTT AUC was calculated. *P< 0.05. Values are reported as mean ± SEM.

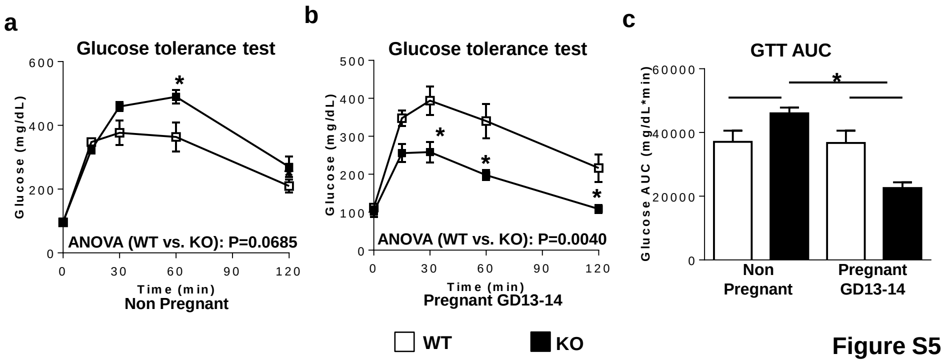

Figure S5

Supplementary Figure 6. Pregnancy does not alter weight of pancreas or β-cell mass or pancreatic insulin content in *SERT*$^{-/-}$ mice. (**a**) Total pancreatic insulin content (n = 6-11 per group) from non-pregnant and pregnant (GD13-14) WT and *SERT*$^{-/-}$ mice at 3-month old. (**b**) Pancreas weight (n = 11-14 per group), (**c**) Representative images of insulin-stained (immunofluorescence, red) pancreatic sections (Scale bar: 100 µm), (**d**) quantification of β-cell mass (n = 3 per group). **P*< 0.05. Values are reported as mean ± SEM.

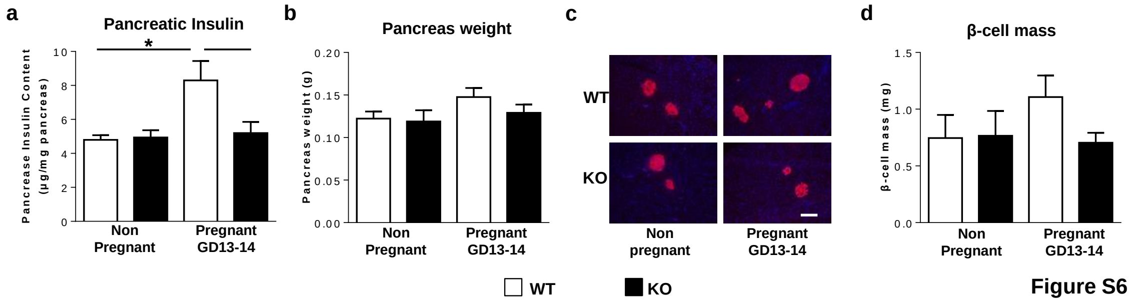

Supplementary Figure 7. Pregnancy increases visceral adipose tissue estrogen receptor expression in *SERT*$^{-/-}$ mice. Esr1 mRNA expression in gWAT was quantified by RT-qPCR, normalized to GAPDH, and expressed relative to the non-pregnant WT group (n = 5 per group). *$P < 0.05$. Values are reported as mean ± SEM.

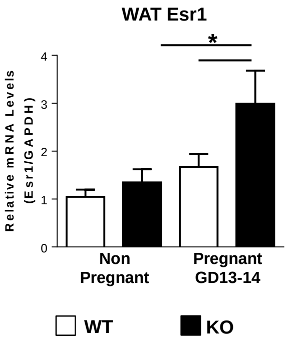

Figure S7

Supplementary Table

Supplementary Table 1. Number of days at diestrus, proestrus, estrus, and metestrus over 14 days of monitoring

**Stage**	**Diestrus**	**Proestrus**	**Estrus**	**Metestrus**
WT	6.5 ± 1.0	2.7 ± 0.3	3.0 ± 0.6	1.8 ± 0.5
KO	14.0 ± 0.0	0.0 ± 0.0	0.0 ± 0.0	0.0 ± 0.0
P Value	<0.05	<0.05	<0.05	<0.05

Values are reported as mean ± SEM. *P* value indicates statistical significance for WT vs KO.

Uncut blot for Figure 2g

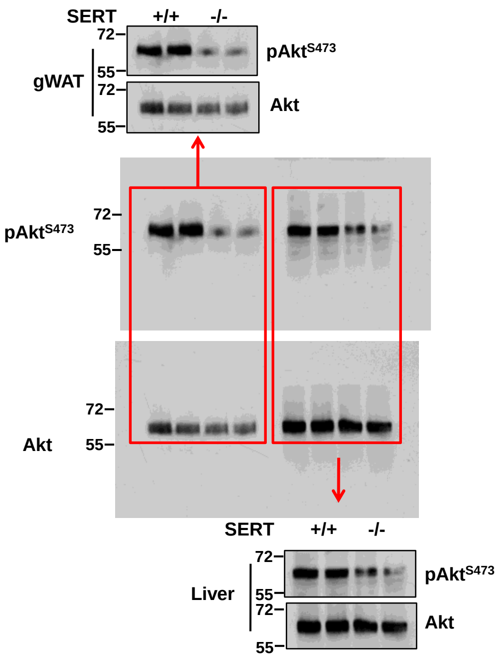

Uncut blot for Figure 3d

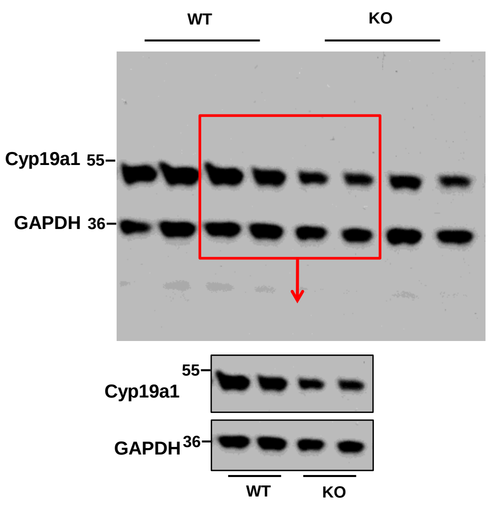

Uncut blot for Figure 4o

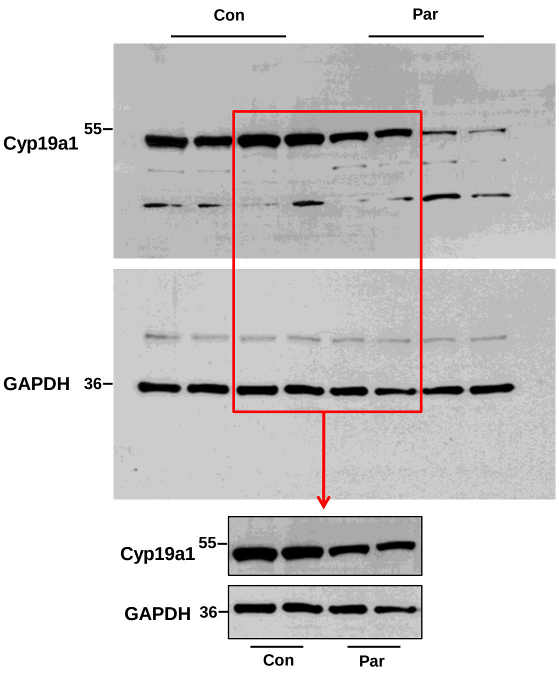

Uncut blot for Figure 5c

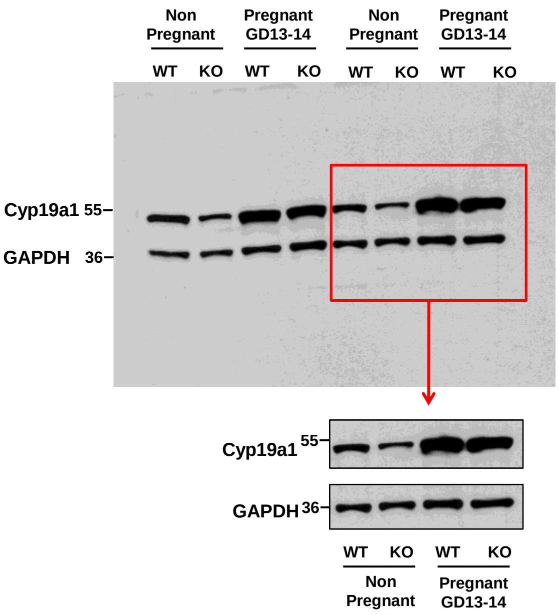

Supplement: Supplementary file 1 — Supplementary Information [file 41598_2017_1291_MOESM1_ESM.pdf]
